# Simultaneously targeting inflammatory response and parasite sequestration in brain to treat Experimental Cerebral Malaria

**DOI:** 10.1038/srep12671

**Published:** 2015-07-31

**Authors:** Chaitanya Dende, Jairam Meena, Perumal Nagarajan, Amulya K. Panda, Pundi N. Rangarajan, Govindarajan Padmanaban

**Affiliations:** 1 Department of Biochemistry, Indian Institute of Science, Bengaluru; 560012; 2 National Institute of Immunology, New Delhi; 110067, India

## Abstract

Malaria afflicts around 200 million people annually, with a mortality number close to 600,000. The mortality rate in Human Cerebral Malaria (HCM) is unacceptably high (15–20%), despite the availability of artemisinin-based therapy. An effective adjunct therapy is urgently needed. Experimental Cerebral Malaria (ECM) in mice manifests many of the neurological features of HCM. Migration of T cells and parasite-infected RBCs (pRBCs) into the brain are both necessary to precipitate the disease. We have been able to simultaneously target both these parameters of ECM. Curcumin alone was able to reverse all the parameters investigated in this study that govern inflammatory responses, CD8^+^ T cell and pRBC sequestration into the brain and blood brain barrier (BBB) breakdown. But the animals eventually died of anemia due to parasite build-up in blood. However, arteether-curcumin (AC) combination therapy even after the onset of symptoms provided complete cure. AC treatment is a promising therapeutic option for HCM.

Although malaria mortality rates have decreased by an impressive 47% between 2000 and 2013, it is still a major affliction of mankind^1^. The failure of a large number of adjunct therapies in HCM^2^ demands the development of new intervention strategies. Experimental Cerebral Malaria (ECM), using *Plasmodium berghei (ANKA)*-infected mice (C57BL/6) manifests many of the neurological features of HCM^3^. In HCM, cytoadherence of pRBCs in brain microvasculature has been implicated as a major contributing factor. While the pathology of HCM is considered as essentially due to sequestration of pRBCs, ECM is essentially due to an inflammatory response^4^,^5^,^6^.

The cellular and pathological features of HCM and ECM were analysed and it was noted that although the differences between the two are recognized, vascular obstruction caused either by pRBCs, leukocytes or both appear to be associated with features of HCM and ECM and the latter model is useful to analyze the relationship between reversal of vessel blockage and inflammation in CM^7^. There is extensive documentation on the association of ECM with sequestration of monocytes and T cells in brain microvessels with attendant up-regulation in the levels of chemokine and chemokine receptors, pro-inflammatory cytokines, adhesion molecules and break down of BBB^3^,^8^,^9^,^10^,^11^,^12^. Studies have also demonstrated the accumulation of pRBCs and CD8^+^ T cells in the brain in ECM and their interdependence to precipitate the disease^8^,^13^,^14^. In a more recent study, it has been clearly shown that the olfactory bulb in the brain is an early target for the parasite, which subsequently spreads to other parts of the brain in the ECM model. There is parasite accumulation and cell occlusion followed by microbleeding, associated with fever and cytokine storm, leading to loss of BBB integrity. These results are also visualized to substantiate the relevance of ECM to study HCM^15^,^16^.

It is important to target the range of functions central to the pathophysiology of both ECM and HCM and this should include parameters governing both pRBC sequestration and leukocyte migration into the brain. It has also been pointed out^7^ that adjunct interventions given before the onset of the disease, while providing information on disease processes in ECM, may not necessarily lead to viable therapies. Therefore, it becomes necessary to study the effect of interventions after onset of the symptoms. Studies in this laboratory had shown that curcumin has antimalarial activity^17^ and AC treatment was very effective in preventing parasite recrudescence in *P. berghei*-infected Swiss mice This was shown as due to suppression of Th1 cytokine response and induction of Th2 cytokine response^18^,^19^. Curcumin loaded chitosan nanoparticles when delivered orally has been shown to protect mice infected with a lethal strain of *P. yoelii*^20^.

It is of interest to study the effect of curcumin and AC treatments in ECM caused by *P. berghei* ANKA in C57BL/6 mice. In the present study, we provide experimental evidence for the first time that curcumin alone, when given after the establishment of infection (3–5 days post-infection), was able to reverse all the inflammatory parameters investigated and pRBC sequestration in the brain, eliminating the symptoms of ECM and delaying the death of mice. AC combination therapy given even after the onset of symptoms provided complete cure by counteracting all the changes characteristic of ECM and preventing parasite build-up in blood and mortality. It has been reported that curcumin, when given orally by gavage during 0–5 days post-infection, delayed the death of mice (C57BL/6) in the ECM model, reversing the symptoms of cerebral malaria^21^.

## Results

### Effect of curcumin on mortality and parasitemia in the ECM model

**Figure 1:**
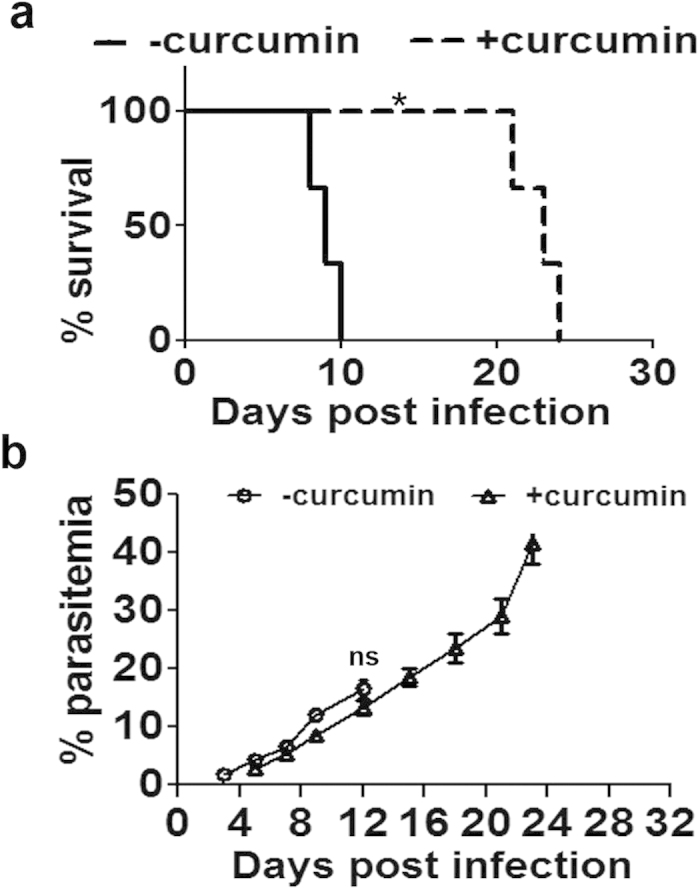
Curcumin prolonged survival of *P.berghei*-infected mice without affecting parasitemia in blood, when given before the onset of neurological symptoms in ECM. **(a)** Curcumin was given in three oral doses at 24 h intervals from day 3 post infection. The data obtained on survival of mice in a typical experiment is provided. Survival curve analysis was carried out by Kaplan-Meier plot and P values calculated by Log-rank (Mantel-Cox) test. **(b)** Parasitemia in blood was assessed using Giemsa stain. The results are from a typical experiment and the values are from five animals for each point. The experiment was repeated thrice. ns, not significant.

C57BL/6 mice were infected with *P. berghei* ANKA through the i.p. route. The infected mice developed typical neurological symptoms between 7 and 10 days post-infection, characterized by inactivity, hind leg paralysis and ataxia, finally ending up with coma and death. The mortality was around 80 to 90%. Two different studies were designed. In the first study, the effect of curcumin alone was studied in the ECM model before the onset of symptoms. In the second study, the effect of AC treatment was studied after the onset of symptoms. The effect on mortality and various parameters governing CD8^+^ T cell and pRBC recruitment in the brain were studied. In the first study, curcumin was given orally at a dose of 5 mg/30 g body weight of the animals on days 3, 4 and 5 after *P. berghei* infection, but before the onset of visible symptoms. The results presented in Fig. 1a indicate that the infected animals died when the parasitemia was around 10–15%, manifesting typical neurological symptoms. This is a characteristic feature reported in other studies as well^4^,^15^. In the present study, curcumin was able to delay death by 15–20 days. Curcumin-treated animals did not show neurological symptoms but died later due to anemia. Initially, curcumin decreased parasitemia in blood marginally which subsequently increased sharply and reached around 40% when the animals died (Fig. 1b).

### Effect of curcumin on BBB and pRBC sequestration

**Figure 2:**
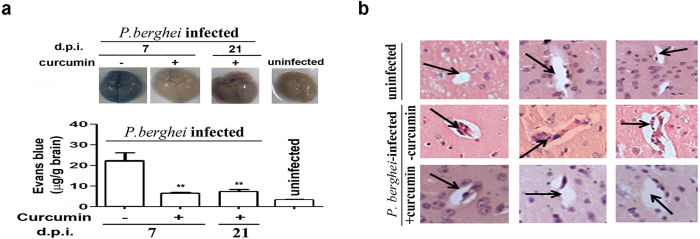
Curcumin suppressed cellular occlusion in brain microvasculature and prevented breakdown of BBB. **(a)** Break down of blood brain barrier was assessed using Evans blue staining *in vivo*. Formamide was used to extract the dye from the brain to measure absorbance. Analysis was carried out on day 7 or day 21 post-infection. The experiment was repeated twice. **(b)** Cellular occlusion in the brain microvasculature was assessed using Hematoxylin-Eosin (H&E) stain. Experiments were carried out using 3 animals per group and brains were processed for H&E staining.

**Figure 3:**
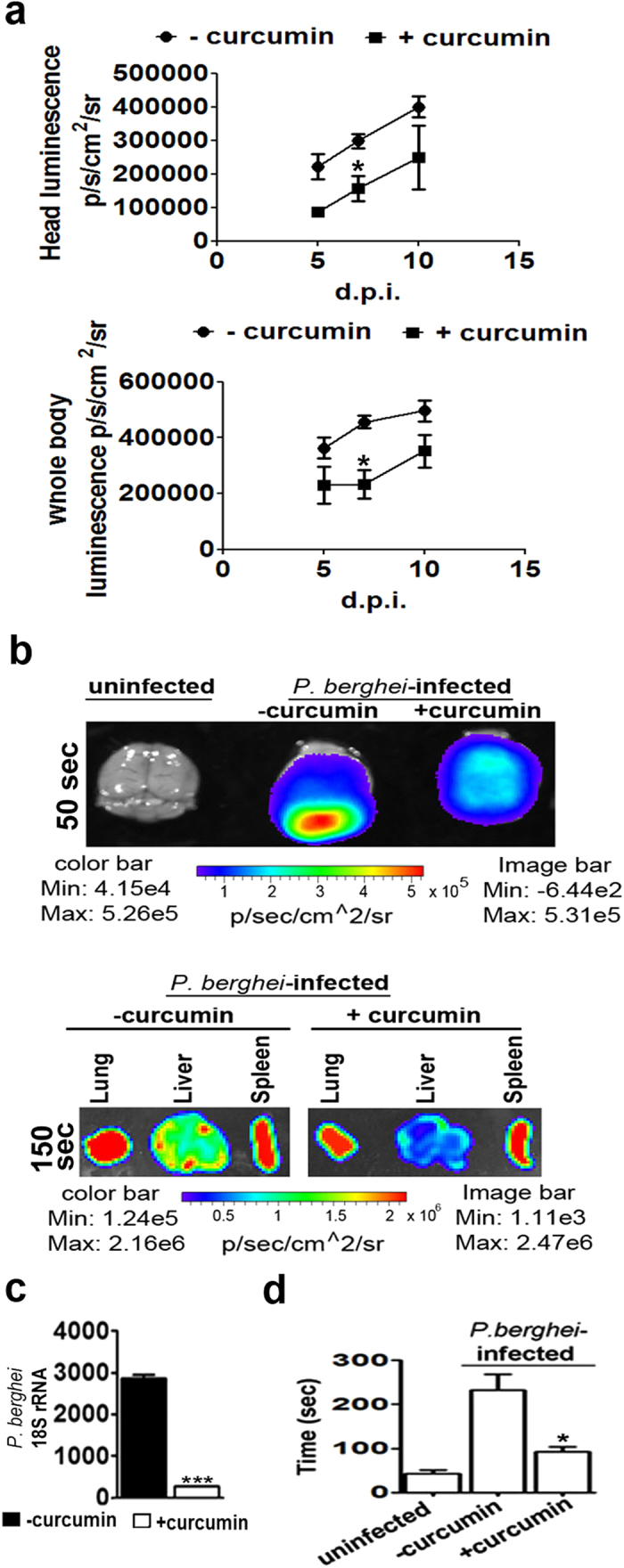
Curcumin suppressed pRBC sequestration into the brain and olfaction loss. **(a)** Whole body imaging of *P. berghei* ANKA transgenic parasites expressing luciferase. On days 5, 7 and 10, whole body and head bioluminescence was recorded after injection of luciferin, 10 min before the imaging. The data represents mean ± SD calculated from three animals in each group. **(b)** *Ex vivo* imaging of brain, liver, lung and spleen dissected from another set of animals sacrificed on day 8 post infection. The numbers on the left side of the panels indicate time of exposure in seconds during image capturing. (**c**) Parasite *18S RNA* in brain. (**d**) Food buried test carried out by recording the time required for the animal to find the buried food. The data represents mean ± SD calculated from four animals in each group. The experiments were repeated twice.

A characteristic feature of the Inflammatory responses in ECM is the breakdown in BBB. Curcumin prevented the breakdown of BBB, as was evident from differential Evans Blue staining of brains of untreated and curcumin-treated *P. berghei*-infected mice (Fig. 2a). Curcumin treatment was also able to prevent cellular occlusion in brain vasculature (Fig. 2b). pRBC sequestration was measured by whole body imaging of mice injected with *P. berghei*-ANKA line expressing luciferase. pRBC sequestration into the brain and other organs was assessed using bioluminescence imaging after injecting luciferin into the mice. Whole body imaging was carried out on days 5, 7 and 10 after infection in addition to obtaining *ex vivo* imaging data separately on day 8. The results presented in Fig. 3a indicate that there was a gradual increase in pRBC sequestration in the head as well as whole body between day 5 to day 10 post-infection. Curcumin was able to significantly inhibit pRBC sequestration at all time points. Representative whole body bioluminescent images are provided in Supplementary Figure 1. Data on the *ex vivo* images of organs, carried out on day 8 with an independent set of animals, also confirmed the inhibitory effects of curcumin on pRBC sequestration into the brain (Fig. 3b). Interestingly, curcumin was able to prevent parasite localization very effectively in liver, but not in lung or spleen (Fig. 3b). Reduction of pRBC sequestration in the brain by curcumin was also reflected in a decrease in parasite 18S rRNA (Fig. 3c). The primers used in qPCR reactions are listed in Supplementary Table 1. The olfaction loss caused by pRBC sequestration, measured using the buried food test^15^,^22^, was also counteracted by curcumin (Fig. 3d).

### Effect of curcumin on parameters of inflammation associated with leukocyte sequestration in the brain

**Figure 4:**
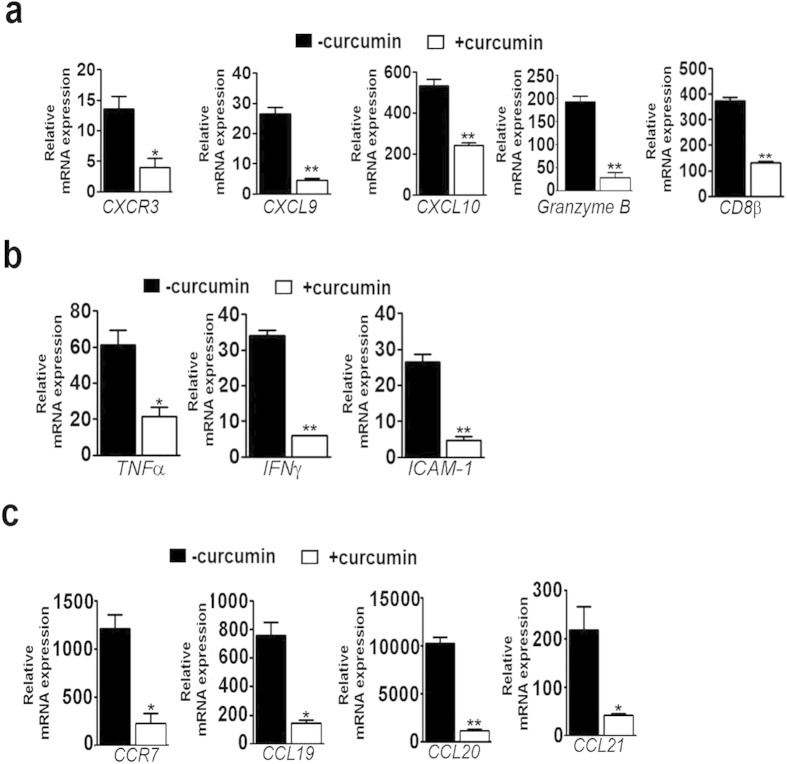
Curcumin suppressed markers of inflammation in the brain. *P. berghei*-infected mice were treated with curcumin as described in Fig. 1 and qPCR analysis was carried out on day 7 post infection from RNA isolated from brain or olfactory bulb. (**a**) *CXCR3*, *CXCL9, CXCL10*, *granzyme B* and CD8β in brain. (**b**) Inflammatory cytokines and cell adhesion molecule in the brain. (**c**) Inflammatory chemokine receptor and its ligands in olfactory bulb. The data are normalized to *18S rRNA* and given as fold changes with respect to RNA from the brain of uninfected animals. The experiments were repeated thrice.

ECM is characterized by inflammatory response leading to up-regulation in the levels of chemokine and chemokine receptors, pro-inflammatory cytokines and adhesion molecules, resulting in BBB breakdown and pRBC sequestration in the brain. The results presented indicate that curcumin effectively blocked the increase in the brain mRNAs encoding the chemokine receptor *CXCR3* and its ligands *CXCL9*, *CXCL10* and granzyme B as well as *CD8β* (Fig. 4a). There was inhibition of the inflammatory cytokines *TNFα* and *IFNγ* as well as the cell adhesion molecule, *ICAM-1* (Fig. 4b). Similar results were obtained with *CCR7* and *CCL21* (Fig.4c), used as markers to evaluate the role of olfactory bulb in ECM^15^. In addition*, CCL19* and *CCL20* behaved the same way.

**Figure 5:**
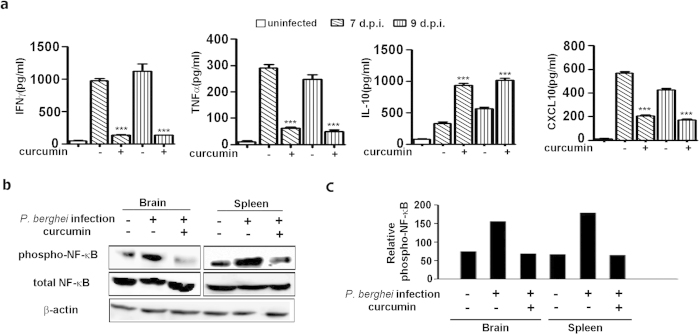
Curcumin modulated the levels of cytokines in serum and suppressed NF-κB activation in brain and spleen. **(a)** Cytokine levels were assessed by ELISA. **(b,c)** Phosphorylated and total NF-κB were assessed by Western blot analysis and data expressed as a ratio in the bar diagram. The data represent mean ± SD from 3 animals in each group. The experiment was repeated twice. Analysis was carried out on day 7 post-infection.

It was of interest to study the effect of curcumin on the levels of serum cytokines. Interestingly, curcumin treatment decreased the serum levels of pro-inflammatory cytokines IFNγ and TNFα, while the level of the anti-inflammatory cytokine IL-10 was enhanced (Fig. 5a). More strikingly, curcumin treatment also resulted in a decrease in serum CXCL10, the only marker claimed to be independently associated with CM mortality among 36 serum biomarkers evaluated in Ghanaian children^23^. The nuclear factor NF-κB plays an important role in the expression of proinflammatory genes. These include chemokines, cytokines and adhesion molecules^24^. Curcumin is a known inhibitor of NF-κB activation in cancer and other types of cells^24^,^25^,^26^. Phosphorylation of NF-κB proteins such as p65 is essential for the induction of target genes^27^,^28^. In the present study, curcumin treatment led to a suppression of phosphorylated p65 in the brain as well as spleen (Fig. 5b). Thus, inhibition of the NF-κB pathway can explain the anti-inflammatory effect of curcumin in the ECM model as well.

**Figure 6:**
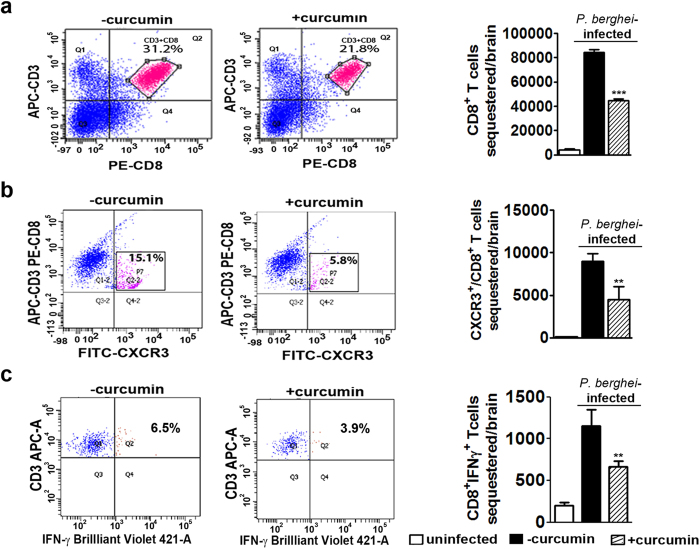
Curcumin suppressed sequestration of activated CD8^+^ T cells in the brain. FACS analysis was carried out to assess sequestration of **(a)** CD3^+^/CD8^+^ cells **(b)** CXCR3^+^/CD3^+^/CD8^+^ cells and **(c)** CD3^+^/CD8^+^/IFNγ^+^ in brain leukocytes. The experiment was repeated twice and two animals were used in each group.

**Figure 7:**
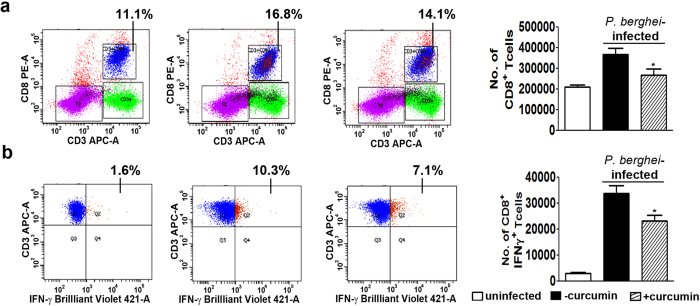
Curcumin suppressed total and activated CD8^+^ T cells in spleen. FACS analysis was carried out to assess **(a)** CD3^+^/CD8^+^ T cells (b) CD3^+^/CD8^+^/IFNγ^+^T cells. The experiment was repeated twice and the data represent mean ± SD from 2 animals in each group.

Apart from studying the effect of curcumin on CD8^+^ T cell marker mRNAs, FACS analysis was also performed to assess sequestration into the brain on day 7. The results presented in Fig. 6a,b and Supplementary Figure 2 indicate that curcumin treatment decreased sequestration of CD3^+^ /CD8^+^ T cells by ~50%. Other studies have also clearly established the requirement of CXCR3 and its ligands CXCL9 and 10 for precipitating ECM^29^ and that CXCR3-deficient CD8^+^ T cells were 7-fold less efficient at migrating into infected brains than wild-type CD8^+^ T cells^30^. FACS analysis indicated that curcumin treatment brought about a reduction in CD3^+^ /CD8^+^ /CXCR3^+^ triple-positive T cells in the brain by ~60%. In addition, since IFNγ has been shown to regulate CD8^+^ T cell migration into the brain^31^, FACS analysis was also carried out to assess sequestration of CD8^+^ /IFNγ^+^ T cells. The results presented in Fig. 6c indicate that once again curcumin treatment reduced CD3^+^ /CD8^+^ /IFNγ^+^ T cells in the brain by 40%. Since, curcumin inhibits phosphorylated NF-κB in brain as well as spleen (Fig. 5b), there is a possibility that curcumin may be inhibiting active CD8^+^ T cell proliferation in the spleen as well. FACS analysis indicates that curcumin treatment inhibited activated CD8^+^ T cell proliferation in spleen by around 35%. Curcumin showed a similar effect on total CD8^+^ T cell proliferation in spleen (Fig. 7).Based on the parameters studied, it can be concluded that curcumin, when given alone before the onset of symptoms was able to significantly inhibit CD8^+^ T cell expansion, leading to a reduction in the sequestration of activated CD8^+^ T cell and pRBC in the brain.

### Effect of combination therapy after the onset of neurological symptoms

**Figure 8:**
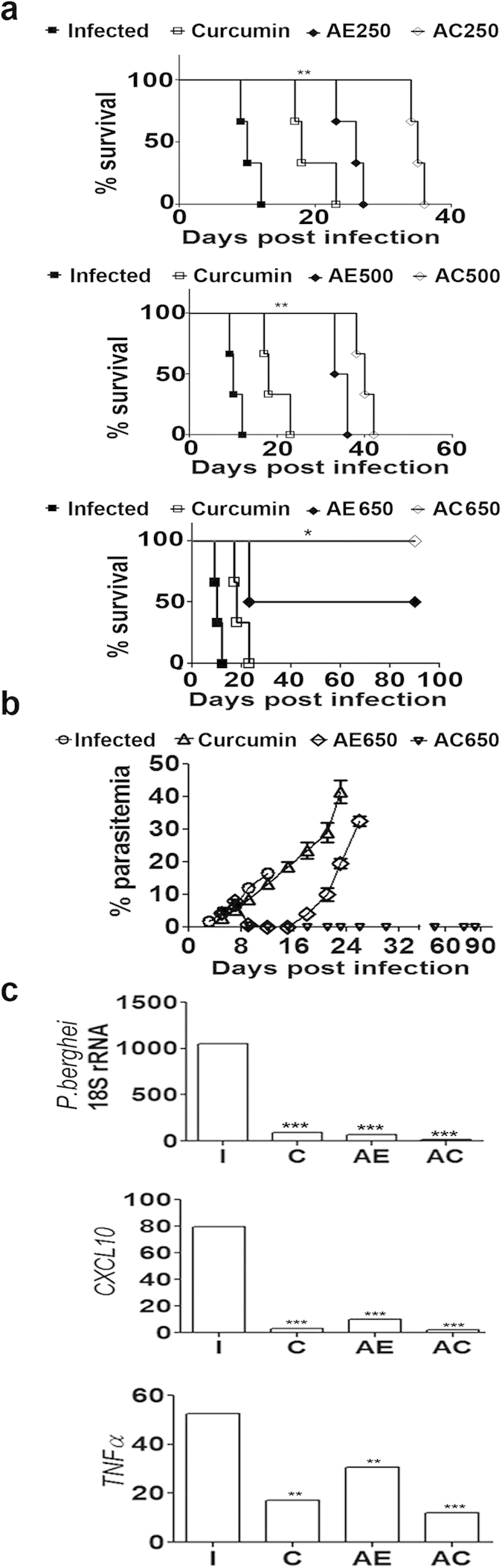
Effect of AC treatment on survival and associated markers after the onset of symptoms of ECM. Curcumin, AE and AC treatments were given beyond 12 h after the onset of symptoms (day 8 or 9 post infection) but before the coma stage. **(a)** Effect of drug treatments on survival of mice. AE was used at different sub-optimal concentrations ranging from 250 μg to 650 μg and given as a single injection. Curcumin was given in 3 oral doses. Survival curve analysis was carried out by Kaplan-Meier plot and P values calculated by Log-rank (Mantel-Cox) test. The experiment was carried out with 5 animals in each group and the experiment repeated twice. **(b)** Parasitemia in blood of the drug treated animals. **(c)** RT-PCR analysis of parasite *18S rRNA*, *CXCL10* and *TNFα* in the brain in the early stage after the drug treatments (day 12, AE 650 μg). The experiment was repeated twice and the data represent mean ± SD from five animals in each group.

**Figure 9:**
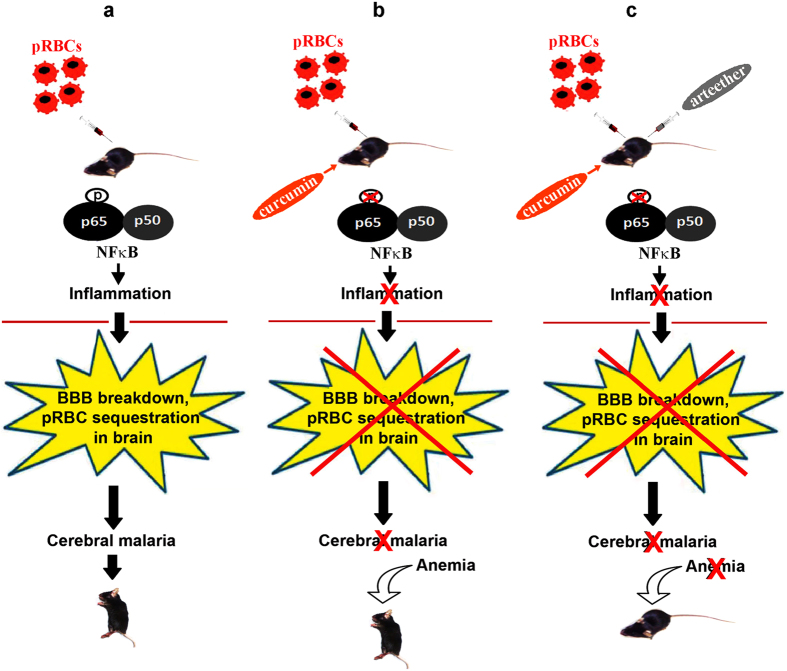
A schematic representation to explain the mechanism of action of curcumin as an adjunct drug to treat Experimental Cerebral Malaria. **(a)** Infection of C57BL/6 mice with *P. berghei* results in phosphorylation NF-κB, disruption of BBB, pRBC sequestration in brain, cerebral malaria and death. **(b)** Curcumin inhibits NF-κB phosphorylation and prevents disruption of BBB and cerebral malaria. However, mice die of anemia due to parasite build-up in blood. **(c)** Co-administration of curcumin and arteether prevents both cerebral malaria and anemia leading to complete protection. The mice shown in the picture were photographed in the laboratory by C.D. The syringes and other illustrations were drawn by P.N.R.

The crucial experiment was to demonstrate the use of curcumin as an adjunct therapy after the onset of neurological symptoms in mice. The animals usually died 24–48 h after the onset of symptoms. AC combination therapy was tested using different concentrations of α,β-arteether (AE, single dose i.m, sub-optimal) and 3 oral doses of curcumin (5 mg/30 g body weight), 12–24 h after the onset of symptoms, but before the onset of coma. Curcumin treatment alone delayed death by 6–8 days. AE treatment alone delayed death by 10–14 days at 250 μg/30 g and 20–26 days at 500 μg/30 g. AC treatment in either case further delayed death by 6–10 days. At 650 μg, AE alone showed around 60% protection. But, AC treatment provided 100% protection against death, for at least 3 months (Fig. 8a). At the time of death, animals treated with curcumin or AE (650 μg) alone showed around ~35–40% parasitemia in blood. AC-treated animals which were completely protected did not show any build-up of parasitemia (Fig. 8b). In the early stage after the onset of neurological symptoms (day 12 post infection), curcumin, AE (650 μg) and AC-treated animals showed inhibition of *P. berghei 18S rRNA*, *CXCL10* and *TNF*α mRNAs (Fig. 8c), similar to the results obtained when curcumin was given before the onset of cerebral malaria symptoms. The decrease in AE treatment is essentially due to the direct killing effect of the drug on the parasite. Thus, curcumin was effective even at suboptimal concentrations of AE to cure ECM. Based on these results, it can be suggested that with a full dose of AE, curcumin would effectively kill the parasite and inhibit inflammatory response. The synergistic action of curcumin with arteether (AC) for the treatment of cerebral malaria is depicted schematically in Fig. 9.

### Bioavailability of curcumin

In view of the concerns regarding bioavailability of curcumin^32^, preliminary studies were carried out using LC-MS/MS to measure curcumin and metabolite concentrations in blood and brain after a single oral dose (5 mg/30 g). The results presented in Supplementary Table 2 indicate that curcumin was detectable at around 3 to 10 ng in blood as well as in brain (ng/g). The values at 2 h were higher than at 4 h in the uninfected animal, but reverse was the case in infected animals. Interestingly, curcumin glucuronide was detected at around 500 ng/g in blood. Thus, curcumin was indeed detectable in blood and brain, undergoing rapid metabolism in blood. Detailed pharmacokinetic studies with the different doses of curcumin would be of interest.

## Discussion

It has been pointed out that a large number of studies with ECM have been targeted at reversing the inflammatory parameters associated with leukocyte migration into the brain and consequences of inflammation. Interventions aimed at prevention of inflammatory responses alone have not succeeded in HCM, since HCM was considered to be primarily due to pRBC sequestration^6^. A consensus seems to have evolved on the basis that both leukocyte migration and pRBC sequestration into the brain are necessary for precipitating HCM and both are interdependent even in ECM^7^,^8^. It has also been pointed that data need to be generated with interventions made after the onset of neurological symptoms rather than in just a prophylaxis mode.

It is of interest to highlight two recent studies in this context. In one, oral administration of vitamin D for 4 days, before or after *P. berghei* infection was shown to protect mice from ECM. While the infected animals showed neurological symptoms between days 5/6, vitamin D-treated animals died between days 11 and 26, depending on the time of initiation of treatment. The animals essentially died of anemia. The protective effect was shown as due to inhibition of strong host inflammatory response, mediated through reduction of dendritic cell response, production of IL-10 and expansion of regulatory T cells (Tregs). Vitamin D therapy was shown to decrease endothelium activation and improve BBB integrity^33^. An earlier study had shown that vitamin D did not confer protection against ECM, when given as three-weekly i.p. injections^34^. While, the differences in results could be due to the dosage and route of administration, data on the effect of vitamin D after the onset of neurological symptoms are not available. Perhaps, a combination therapy with vitamin D and antimalarials such as artemisin-derivatives could be examined.

Another study has reported the efficacy of atorvastatin-artemether combination therapy in ECM^35^. The main basis for the use of atorvastatin, a cholesterol-lowering agent, is its ability to lower the levels of the chemokine CXCL10. An earlier study had shown that atorvastatin alone had no effect on survival of mice in the ECM model, but had a prophylactic effect in delaying mortality and onset of neurological symptoms, when given in conjunction with mefloquine^36^. It has been pointed out that this combination did not reverse established CM pathologies observed in clinical practice^35^. In the study using atorvastatin-artemether combination, it has been shown that this therapy given for 4 days (day 6–9) after the onset of neurological symptoms in mice, prolonged survival for at least 21 days (100%), with artemether- and atorvastatin-alone treatments providing 75% and 50% survival, respectively. The blood parasitemia values recorded on day 15 were around 20% and 8% and 0% for atorvastatin, artemether and atorvastatin+artemether treatments, respectively^35^. It is claimed that the superiority of atorvastatin over other statins, which are ineffective, is the specific effect of the former in decreasing CXCL10 or its mRNA levels in serum and brain, with a corresponding decrease in CXCL10-mediated network components^35^. As already pointed out, there is evidence of elevated serum levels of CXCL10 to serve as a marker for fatal HCM^23^,^37^ and its role in leukocyte migration into the brain in ECM has been demonstarted^29^,^30^.

The potential of curcumin to reduce CD8^+^ T cell as well as pRBC sequestration in the brain would render it as an ideal adjunct drug to prevent and treat cerebral malaria. In the present study, curcumin alone was found to be effective in reducing leukocyte as well as pRBC sequestration in the brain, maintaining BBB integrity, as revealed by proinflammatory cytokine markers and imaging data. However, curcumin alone could only delay the death of the animal, since it is only moderately effective in preventing mortality as compared to arteether^17^,^18^,^19^. While, it can prevent pRBC sequestration in the brain, parasitemia builds up in blood and the animals die due to anemia and not CM. However the curcumin-artether combination, given after the onset of neurological symptoms, was very effective in counteracting all the investigated parameters governing leukocyte as well as pRBC sequestration. All the animals were protected against mortality at least for 90 days, when an appropriate single sub-optimal dose of arteether and 3 oral doses of curcumin were used. There was no parasite build-up in blood. Thus, we propose that a full dose of arteethter, which translates to multiple doses (i.v) of artesunate or artemether used in clinical practice in the human, along with 3 oral doses of curcumin, could prove beneficial in the treatment of HCM. It is understood that HCM develops through complex mechanisms in different patients, leading to brain injury and coma. It has been suggested that combinations of adjuvant therapies may be needed to target specific mechanisms to improve neuro-cognitive outcomes, since HCM afflicts an unacceptable number of children^38^. It is in this context that a curcumin-artemisinin derivative combination therapy can be very effective.

Earlier studies in this laboratory have demonstrated the efficacy of curcumin-based combination therapy to prevent parasite recrudescence in uncomplicated malaria model in Swiss mice that led to proposals for the study of this combination in cerebral malaria^17^,^18^,^19^,^39^,^40^,^41^. The present study establishes the efficacy of curcumin as an adjunct drug in the treatment of ECM. Curcumin as a dietary component is superior to atorvastatin or any other drug in view of the absence of side effects and resistance development, in addition to its lower costs. In a phase I clinical trial, curcumin was given at a dose of 8 g per day for 3 months without side effects^42^.

An issue with curcumin is its reported poor bioavailability^32^, In many studies, the stability of the molecule in serum/plasma or tissue during processing has not been ensured and this has been highlighted^43^. Preliminary data indicate that curcumin is detectable in plasma and brain even after a single oral dose. The main issue appears to be its rapid metabolism to glucuronide. Similar results were reported in humans after a single oral dose, where curcumin conjugates were detected, indicating that curcumin is indeed absorbed^44^. While the contribution of curcumin conjugates to the results obtained needs to be studied, it is worth investigating the use of nanocurcumin as an adjunct drug in this context.

## Methods

### Ethics statement

Animal experiments were carried out as per the guidelines of the Committee for the Purpose of Control and Supervision of Experimental Animals (CPCSEA), Government of India (Registration No: 48/1999/CPCSEA) and as approved by the Institutional Animal Ethics Committee (IAEC) of the Indian Institute of Science, Bangalore (CAF/Ethics/102/2007-435 and CAF/Ethics/192/2010). C57BL/6J animals (maintained at the Indian Institute of Science, Bangalore, India and National Institute of Immunology, New Delhi, India) were used as a well characterized animal model for murine cerebral malaria.

### Infection and drug treatment

*P. berghei* ANKA strain (MRA-311, MR4, ATCC Manassas Virginia) was maintained in 6–8 weeks old C57BL/6 mice, which were infected with 3 × 10^6^ parasitized red blood cells (pRBC). The animals were checked every day for symptoms and parasitemia using Giemsa stain. 80–90% of the *P. berghei* ANKA infected animals showed typical cerebral malaria symptoms, which included deviation of head, ruffled hair, hind limb paralysis, ataxia, convulsions and coma as reported^30^ between days 7–10 after infection. Animals were treated from day 3 post infection (before onset of symptoms) or from day 8/9 post infection (after onset of symptoms) with Curcumin (5 mg/mouse in DMSO). Three oral doses were given at 24 hr intervals. AE was given as a single injection (i.m.) after onset of symptoms in the concentration range 250–650 μg/animal. Blood stage parasite *P. berghei* ANKA and *P. berghei* ANKA line expressing green fluorescent protein GFP-Luciferase construct were cryopreserved in liquid nitrogen.

### Blood-brain barrier permeability

Blood-brain barrier permeability was checked by Evans blue assay. Briefly, mice were anesthetized and given intravenous injection of 0.2 ml of 2% Evans blue dye in PBS. After 2h, the blue dye was extracted from the brain tissue with 100% formamide and absorbance measured at 620 nm^8^.

### qPCR analysis

RNA was isolated from mouse brain/olfactory bulb by TRI Reagent SIGMA-ALDRICH. 1μg of RNA was taken for reverse transcription reaction using random hexamers. The prepared cDNA was diluted 1:25 times and used for qPCR. Real time PCR was carried out using ABI StepOnePlus^TM^ System. Housekeeping gene *18S rRNA* was used as an endogenous control. *CXCR3, CXCL9, CXCL10, ICAM1, TNFα, IFNγ* and *CCR7, CCL19, CCL20, CCL21*, *CD8β*, *granzyme* and parasite *18s rRNA*, transcripts were quantified in the brain and olfactory bulb, respectively, using primers listed in Supplemetary Table 1. The relative gene expression levels were calculated by 2^–ΔΔCT^ method.

### Cytokine and chemokine detection

IL-10 and IFNγ cytokine levels were measured in 20 μl of mouse serum using Murine ELISA kits from BD OptEIA^™^ as per the manufacturer’s protocol. Biotinylated Detection Antibody and Avidin-HRP conjugate were used with 3′, 3′, 5, 5′ Tetramethylbenzidine (TMB) liquid substrate to give a colored product. Reaction was stopped using 2N H_2_SO_4_. A_450_ was measured using the Infinite 200 PRO TECAN microplate reader. CXCL10 chemokine, TNFα cytokine levels were detected using Krishgen biosystems ELISA kits.

### Phospho NF-κB estimation

Whole brain was homogenized in 0.1 M potassium phosphate buffer containing 0.25M sucrose in the presence of protease inhibitors (Roche)^45^. Brain homogenate was centrifuged at 14000 g for 20 minutes to obtain brain lysate. Spleen was homogenized in RIPA buffer and processed similarly. The lysates (50 μg/lane) were analysed on SDS-PAGE and Western blot carried out with p-NF-κB antibody (1:1000, CST Rabbit monoclonal #3033) anti-rabbit HRP conjugate. The blots were developed using ECL substrate (Millipore), ImageQuant LAS 4000 machine.

### Flowcytometry of brain sequestered leukocytes

Brains were collected from uninfected as well as untreated and curcumin-treated *P. berghei*-infectedC57BL/6 mice, 7 days post infection, after perfusion of the animal intracardially with PBS. Brains were immediately placed in DMEM medium containing 10% serum. Brain tissue was cut into pieces and enzymatically digested with 0.05% collagenase D (Roche) and 2U/ml DNase I (Thermo scientific) for 20 min at 37 ^o^C^29^. Tissue extracts were sieved using 70 μm nylon mesh, and the supernatant, free of cellular debris was centrifuged at 400 xg for 5 min and pellet was re-suspended in DMEM medium and layered on 30% (v/v) percoll (Sigma Aldrich) centrifuged at 400× g for 20 min. The cell suspension was treated with RBC lysis buffer (155 mM NH_4_Cl, 10 mM NaHCO_3_, 0.1 mM EDTA, pH 7.3) to remove RBCs. Cell viability was checked using Trypan blue (Sigma Aldrich). Viable cells were counted and 10 × 10^6^ cells were used for flow cytometry. Purified cell suspensions were treated with 0.5% BSA and 10% FCS for 30 min, followed by treatment with APC-conjugated, anti-mouse CD3e antibody (clone145-2C11, BD Biosciences), FITC-conjugated, anti-CXCR3/CD183 antibody (clone CXCR3-173, eBioscience) and PE-conjugated, mouse anti-CD8a antibody (clone53-6.7, BD Biosciences) for 1 h at 4 °C. Cells were washed with DMEM and fixed with 4% paraformaldehyde followed by PBS wash. Cells were finally suspended in PBS and used for FACS analysis in BD FACS Canto^™^ II machine. Day 7 or Day 8 post infection, intracellular cytokine (IFNγ) staining was carried out by isolating lymphocytes from brain or Splenocytes of the Infected or curcumin treated animals. Cytokine secretion was blocked by protein transport inhibitor Brefeldin A (Sigma) at a final concentration of 10 μg/ml for 4 h. Cell surface antigen staining was first performed with anti-CD3 and anti-CD8 antibodies and the cells were fixed with 4% PFA in the dark for 20 min at room temperature. The cells were then incubated with BV421-conjugated, anti-IFNγ antibody (clone XMG 1.2) (BD Biosciences). Cells were washed with PBS and used for FACS. Results were analyzed using BD FACS DIVA^™^ software.

### *In vivo* imaging

pRBC sequestration was measured by whole body imaging of mice injected with *P. berghei*-ANKA line expressing luciferase essentially as described by Claser *et al*.^46^. The bioluminescence images were visualized by an intensified-charge-coupled device (I-CCD) photon-counting video camera from the *in vivo* Imaging System IVIS Lumina 200 (Xenogen)^8^. C57BL/6 mice were infected with 3 × 10^6^ *P. berghei* ANKA transgenic parasite line expressing GFP-Luciferase. On the specified days post infection, animals were injected intraperitoneally with D-Luciferin substrate (promega, VivoGlo^™^) and checked for bioluminescence signal after 10 min in the whole body and head of animals. Whole body and head imaging were carried out with 21.8 and 4 cm FOV, respectively and medium binning factor. The exposure time was varied between 50 and 300 sec depending on the intensity of the signal. In another set of experiments, animals were sacrificed, whole body perfusion performed, brains and other organs were removed and used for *ex vivo* imaging. Average radiance (p/sec/cm^2^/sr) values were calculated for region of interest (ROI) using Living Image®4.3.1(64 bit) software for experimental and control animals. Background values obtained from uninfected mice injected with luciferin were subtracted and plotted using GraphPad Prism 5 software.

### Histology

To make paraffin sections of the brain, whole body perfusion was carried out using PBS followed by 4% paraformaldehyde. The brains were fixed in 4% PFA overnight. Dehydration was carried out using sequential alcohol washes with 30%, 50%, 70%, 90%, 100% ethanol. Xylene was used to perforate brain tissue. Brains were moulded using paraffin wax. Five micron tissue sections were made and collected on poly L- Lysine coated slides and stained with haematoxylin and eosin^8^. Slides were checked under light microscope using AxioVision software to capture images.

### Buried Food Test

This was carried out as described by Yang and Crawley^30^. Briefly, 3 oral doses of curcumin were given starting 5 days post infection and the mice were starved for 24 h before the time taken by the animal to find the food buried under the bedding was recorded.

### Estimation of curcuminoids in plasma and brain

Samples were processed immediately or stored in liquid nitrogen for storage. Plasma was weighed and loaded on a SPE column cartridge which was prior activated with 1.5 ml of 0.05% acetic acid in methanol and washed with 1.5 ml of 0.05% acetic acid in water. After sample loading, the SPE tubes (Strata-X 33 μ polymeric reversed phase; 8B-S100-UBJ Phenomenex) were washed with 1.5 ml of 0.05% acetic acid in water and the analytes eluted with 4 × 1 ml of methanol: acetonitrile (1:1). The organic phase was evaporated to dryness using high performance personal evaporating system (Genevac UK). Brain samples were powdered after adding liquid nitrogen, then extracted with ethyl acetate: methanol: acetic acid (95:5:0.05) and the solution was centrifuged. The supernatant was evaporated to dryness as before. Pooled samples were taken for analysis. An LC system (Waters, Corporation, Milford, U.S.A) consisting of an Acquity ultra Performance LC and electrospray chemical ionization tandem mass spectrometer (ESCI-MS/MS; Waters) was used. The samples were separated on Ambient BEH C18 (2.1 × 50 mm), 1.7 μ or Equivalent L1 column. The calibration curves of bisdemethoxy curcumin (BDMC), demethoxy curcumin (DMC) and curcumin were linear over the concentration range of 1–800 ppb. The LOQ for curcumin, demethoxy curcumin and bisdemethoxy curcumin were 10 ppb. The plasma and tissue sample residue was reconstituted with an appropriate volume of acetonitrile:water containing 0.1% formic acid (1:1) and transferred into a micro-vial. A 5 μl aliquot was injected into LC-MS/MS system and analyzed for curcuminoids and metabolites. Data acquisition and quantitation were performed using MassLynx software version 4.1. These analyses were carried out at Arjuna Natural Extracts, Cochin, India.

### Statistical analysis

Statistical tests were carried out by One-way analysis of variance (Bonferroni’s Multiple Comparison Test) or by Unpaired two tailed t test using GraphPad Prism 5. Data are presented as mean ± S.D. P value summary is mentioned on the bar of the each figure. *P < 0.05; **P < 0.001; ***P < 0.0001. ns, not significant. Survival curve analysis was carried out by Kaplan-Meier plot and P values calculated by Log-rank (Mantel-Cox) test.

## Additional Information

**How to cite this article**: Dende, C. *et al*. Simultaneously targeting inflammatory response and parasite sequestration in brain to treat Experimental Cerebral Malaria. *Sci. Rep*. **5**, 12671; doi: 10.1038/srep12671 (2015).

## Supplementary Material

Supplementary Information
