# Supplementary material for: Simultaneously targeting inflammatory response and parasite sequestration in brain to treat Experimental Cerebral Malaria
Source: Sci Rep. 2015 Jul 31;5:12671. doi: 10.1038/srep12671 (PMC4521148; doi:10.1038/srep12671)
Supplement: Supplementary Information [file srep12671-s1.pdf]

Supplementary information for:

Manuscript title: **Simultaneously targeting inflammatory response and parasite sequestration in brain to treat Experimental Cerebral Malaria**

Chaitanya Dende[1], Jairam Meena[2], Perumal Nagarajan[2], Amulya K Panda[2], Pundi N Rangarajan[1], Govindarajan Padmanaban[1].

[1]Department of Biochemistry, Indian Institute of Science, Bengaluru-560012 and [2]National Institute of Immunology, New Delhi.

Correspondence to be addressed to: P.N.R. (pnr@biochem.iisc.ernet.in) or G.P. (geepee@biochem.iisc.ernet.in).

Supplementary Figure 1. Representative whole body bioluminescent images of untreated and curcumin-treated mice infected with *P. berghei*-ANKA transgenic parasites expressing luciferase.

Supplementary Figure 2. FACS analysis of $CXCR3^+/CD3^+/CD8^+$ cells in brain leucocytes of uninfected and *P. berghei*-infected mice.

Supplementary Table 1. Primers used in qPCR reactions

Supplementary Table 2. Analysis of curcumin in plasma and brain of mice.

Supplementary Figure 3. Unprocessed original scans of blots in Figure 5b

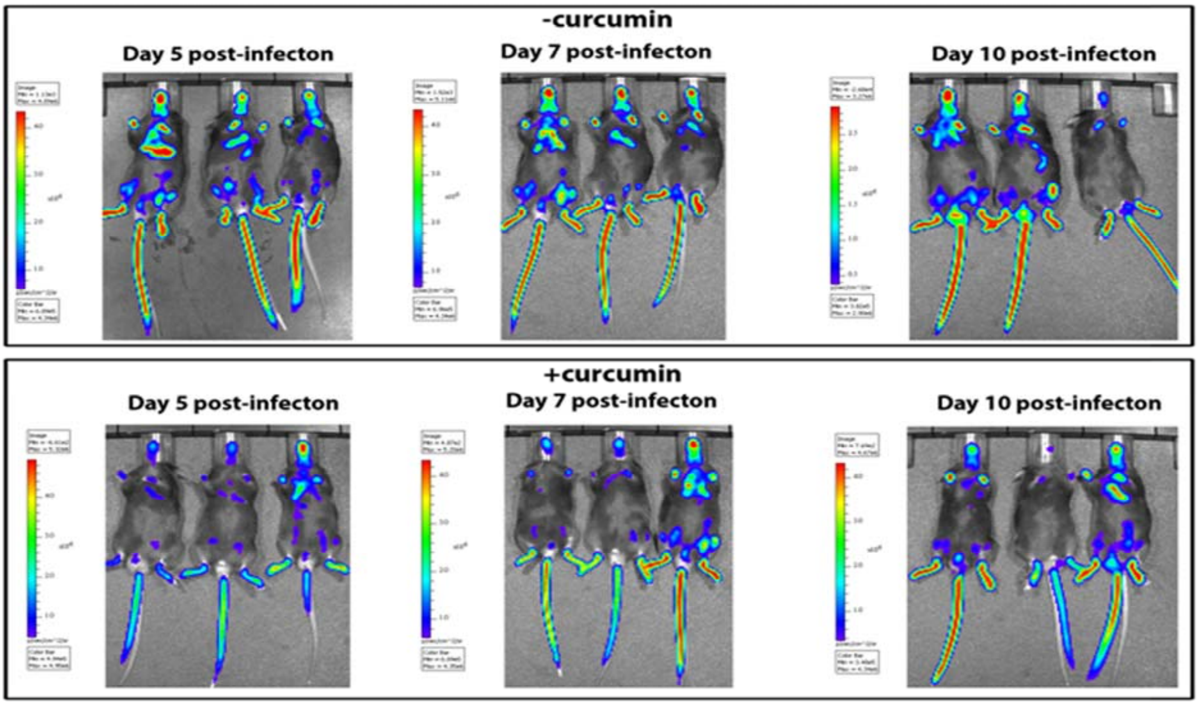

Supplementary Figure 1. Representative whole body bioluminescent images of untreated (-curcumin) and curcumin-treated mice infected with *P. berghei*-ANKA transgenic parasites expressing luciferase.

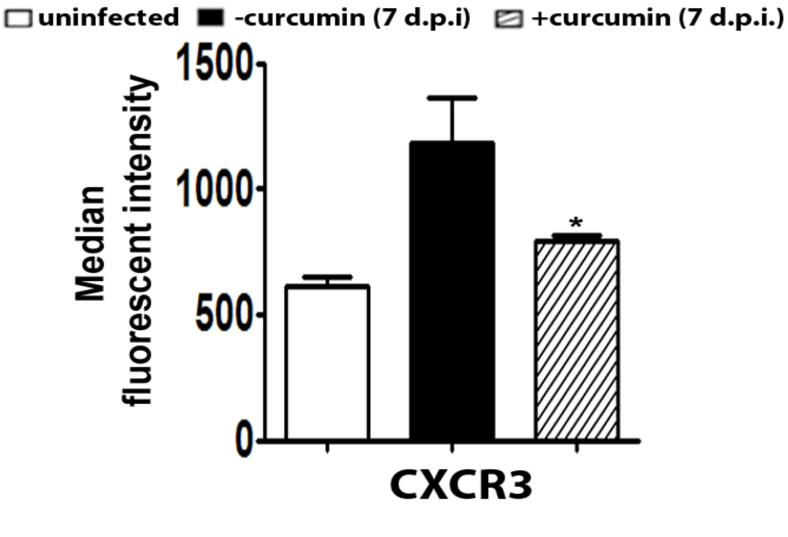

Supplementary Figure 2. FACS analysis of $CXCR3^+/CD3^+/CD8^+$ cells in brain leucocytes of uninfected and *P. berghei*-infected mice.

Supplementary Table 1. Primers used in qPCR reactions

Gene	Gene sequence
CXCR3	Forward 5'AATGCCACCCATTGCCAGTAC 3'<br>Reverse 5' AGCAGTAGGCCATGACCAGAAG 3'
CXCL9	Forward 5' GCCATGAAGTCCGCTGTTCT 3'<br>Reverse 5' GGGTTCCTCGAACTCCACACT
CXCL10	Forward 5' GACGGTCCGCTGCAACTG 3'<br>Reverse 5' GCTTCCCTATGGCCCTCATT 3'
TNFα	Forward 5' AAGCCTGTAGCCCACGTCGTA3'<br>Reverse 5' GGCACCACTAGTTGGTTGTCTTTG 3'
IFNγ	Forward 5' TCAAGTGGCATAGATGTGGAAGAA<br>Reverse 5' TGGCTCTGCAGGATTTTCATG 3'
ICAM1	Forward 5' GCCTCCGGACTTTCGATCTT 3'<br>Reverse 5' GTCAGGGGTGTCGAGCTTTG 3'
CD8β	Forward 5' GCTGGTAGTCTGCATCCTGCTTC 3'<br>Reverse 5' TTGCTAGCAGGCTATCAGTCTTGTG 3'
18S rRNA P. berghei ANKA	Forward 5' AAGCATTAAATAAAGCGAATACATCCTTAC 3'<br>Reverse 5' GGAGATTGGTTTTGACGTTTATGTG 3'
CCR7	Forward 5' TCATTGCCGTGGTGGTAGTCTTCA 3'<br>Reverse 5' ATGTTGAGCTGCTTGCTGGTTTCG 3'
CCL19	Forward 5' ATGTGAATCACTCTGGCCCAGGAA 3'<br>Reverse 5' AAGCGGCTTTATTGGAAGCTCTGC 3'
CCL20	Forward 5' CGACTGTTGCCTCTCGTACA 3'<br>Reverse 5' GAGGAGGTTCACAGCCCTTT 3'
CCL21	Forward 5' TGAGCTATGTGCAAACCCTGAGGA3'<br>Reverse 5' TGAGGGCTGTGTCTGTTCAGTTCT3'
Granzyme B	Forward 5' CCTCCTGCTACTGCTGAC 3'<br>Reverse 5' GTCAGCACAAAGTCCTCTC 3'

Supplementary Table 2. Analysis of curcumin in plasma and brain of mice.

Sample	Curcumin (ng/g)	DMC (ng/g)	BDMC (ng/g)	Curcumin glucuronide (ng/g)
Uninfected 2h plasma	2.84	ND	ND	541.32
Uninfected 4h plasma	11.86	4.86	ND	420.16
Infected 2h plasma	10.96	1.78	0.3	525.7
Infected 4h plasma	2.88	0.28	0.28	51.94
Uninfected 2h brain	1.97	0.25	0.082	ND
Uninfected 4h brain	1.60	0.084	ND	ND
Infected 2h brain	4.77	0.59	0.17	ND
Infected 4h brain	ND	ND	ND	ND

ND, Not detected; DMC, demethoxy curcumin; BDMC, bisdemethoxy curcumin

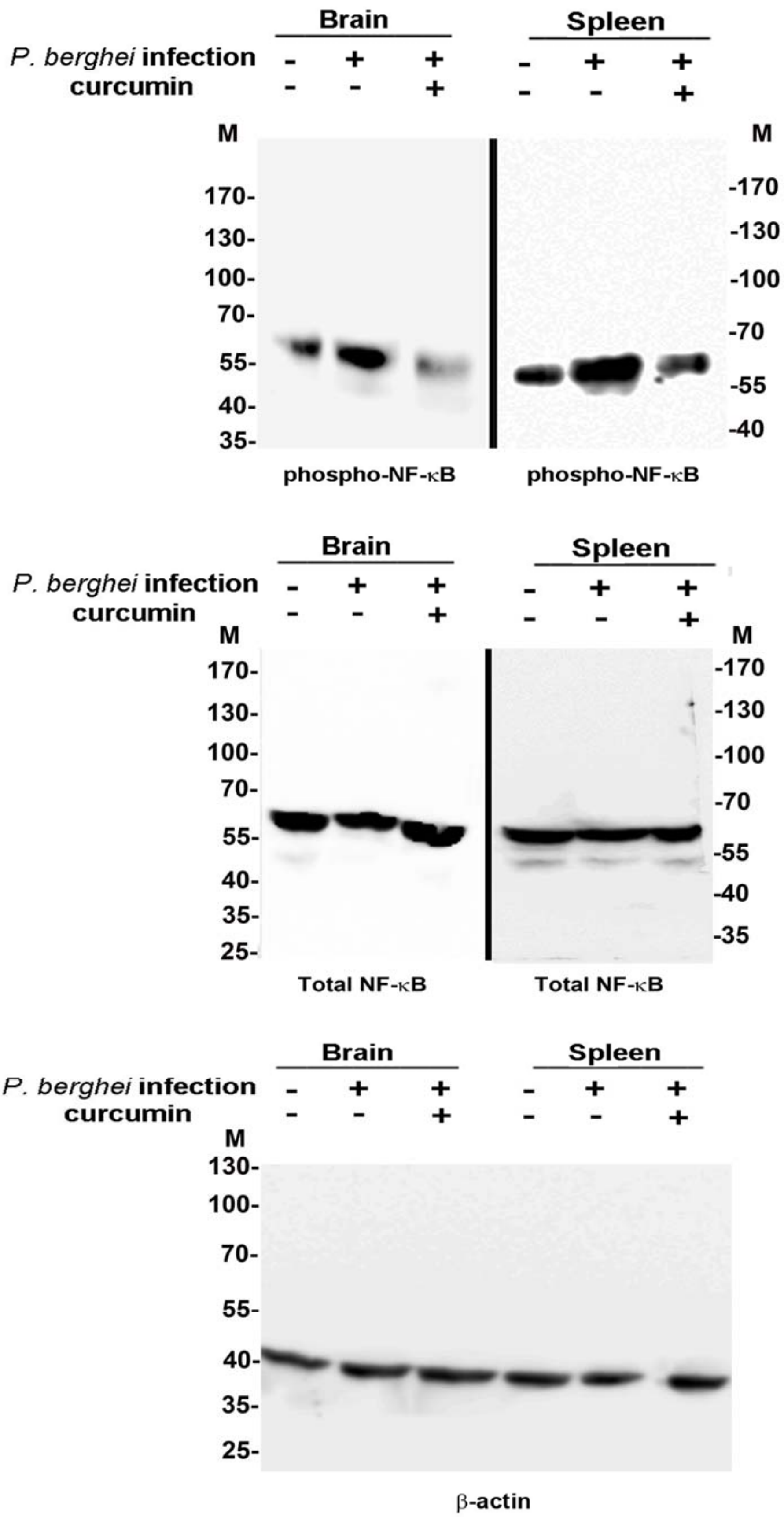

Supplementary Figure 3. Unprocessed original scans of blots in Figure 5b.
